# A Prospective Study on the Metabolic and Hormonal Outcomes of SGLT2 Inhibitor Combination Therapy With and Without Metformin in Newly Diagnosed Type 2 Diabetes Mellitus

**DOI:** 10.3390/biomedicines14061201

**Published:** 2026-05-27

**Authors:** Rahima Aftab, Asher Fawwad, Eraj Abbas, Ruqaya Nangrejo, Fasiha Fatima, Syed M. Shahid

**Affiliations:** 1 Department of Biochemistry, Baqai Medical University, Karachi 75340, Pakistan; rahimaaftab333@gmail.com (R.A.); asherfawwad@baqai.edu.pk (A.F.); 2 Department of Physiology, Baqai Medical University, Karachi 75340, Pakistan; ruqayakhan@baqai.edu.pk; 3 Department of Biochemistry, Karachi Institute of Medical Sciences, Karachi 75080, Pakistan; fasiha.fatima@gmail.com; 4 School of Health & Sport Science, Eastern Institute of Technology (EIT), Auckland Campus, Auckland 1010, New Zealand

**Keywords:** type 2 diabetes mellitus, hypoglycemic agents, SGLT2 inhibitors, metformin, combination

## Abstract

**Background/Objectives:** The rising global burden of type 2 diabetes mellitus (T2DM) demands multifaceted and more effective treatment strategies beyond monotherapy to achieve optimal metabolic control. The study aimed to evaluate the integrated effects of SGLT2 inhibitors and metformin in newly diagnosed T2DM patients on biochemical parameters, clinical outcomes and hormonal changes. **Methods**: This prospective longitudinal study was conducted at the Department of Biochemistry, Baqai Medical University, in collaboration with the Baqai Institute of Diabetology and Endocrinology. A total of 120 newly diagnosed T2DM patients were enrolled and stratified into three groups (n = 40): Group 1 (SGLT2 inhibitors only), Group 2 (SGLT2 inhibitors + metformin), and Group 3 (metformin only). Patients were followed for six months with data collection at baseline, at 3 months and 6 months. Anthropometric indices (weight, BMI, waist and hip circumferences, WHR), biochemical markers (FBS, HbA1c, lipid profile, uric acid, serum creatinine, HOMA-IR), and hormonal levels (insulin, glucagon) were assessed at baseline, first follow-up, and second follow-up. ANOVA, post hoc, Bonferroni and Tukey’s tests were applied; *p*-value < 0.05 was considered significant. **Results**: The findings indicate that Group 2 showed the greatest improvement in anthropometric parameters, particularly waist and hip circumferences (*p* < 0.01). Group 3 demonstrated the most significant improvement in glycemic indices and lipid profile (*p* < 0.01). HOMA-IR significantly decreased in Group 3 from baseline to the first follow-up (*p* < 0.01). While insulin levels remain insignificantly different among all groups. Glucagon levels declined significantly from baseline to the second follow-up in all groups, with a more pronounced decrease in Group 3 (*p* < 0.01). Serum creatinine and uric acid levels showed significant reductions from baseline to the second follow-up in Group 1 and Group 2 (*p* < 0.05). However, given the observational design, these associations should not be interpreted as causal evidence of renoprotection. **Conclusions**: Within the limitations of this observational study, early differences among treatment regimens were observed, though metabolic outcomes became statistically comparable across groups by six months. These hypothesis-generating findings suggest potential benefits of early combination therapy that require confirmation in randomized controlled trials. Given the substantial within-group variability and non-randomized design, no definitive conclusions about therapeutic associations can be drawn from these data.

## 1. Introduction

Chronic hyperglycemia, insulin resistance, and a progressive loss of pancreatic β-cell function are the main characteristics of type 2 diabetes mellitus (T2DM), a complicated metabolic condition that is a fast-growing global health concern [1]. An estimated 589 million adults aged 20–79 years are currently living with diabetes worldwide, representing approximately 1 in 9 individuals in this age group. This burden is projected to increase substantially, reaching 853 million by 2050 [2].

Numerous microvascular (retinopathy, nephropathy, neuropathy) and macrovascular (cardiovascular and cerebrovascular) problems are linked to chronic hyperglycemia in diabetes. With yearly treatment costs reaching $170 billion, these sequelae have a substantial impact on morbidity, mortality, and healthcare expenses [3]. Biguanides, sulfonylureas, thiazolidinediones, and DPP-4 inhibitors are examples of conventional oral hypoglycemic medications that mainly work by improving peripheral insulin sensitivity or increasing insulin secretion through insulin-dependent mechanisms [4]. These drugs are widely used; however, their long-term effectiveness in preserving pancreatic β-cell function may be limited. In addition, they have been associated with certain adverse effects, including hypoglycemia, weight gain, gastrointestinal intolerance, and potential impacts on renal function [5].

A paradigm shift in the treatment of diabetes is represented by sodium-glucose co-transporter-2 (SGLT2) inhibitors. SGLT2 inhibitors increase urine glucose excretion and decrease plasma glucose levels by selectively blocking glucose and salt reabsorption in the proximal renal tubules through an insulin-independent mechanism [6]. By simulating a calorie deficit, the induced glucosuria encourages beneficial metabolic adaptations, such as little weight loss, lower blood pressure, and better cardiovascular and renal outcomes. Because of this, SGLT2 inhibitors have a variety of therapeutic benefits and are being used more frequently in modern type 2 diabetes treatment plans [7].

In addition to being well-tolerated, SGLT2 inhibitors work well in combination with a variety of antidiabetic medications, such as metformin, to target different pathophysiological aspects of type 2 diabetes [8]. Furthermore, the pleiotropic effects of SGLT2 inhibitors, including possible cardioprotective activities, have attracted interest. Evidence indicates that SGLT2 inhibition can considerably lower the risk of hospitalization for heart failure in both diabetics and non-diabetics. Cardiovascular disease continues to be the primary cause of death for people with diabetes [9,10]; these results are changing the paradigm for managing diabetes in favor of a more all-encompassing, organ-protective strategy.

Because of its intricacy and the fact that treatment outcomes might differ from person to person, managing type 2 diabetes is difficult. Therefore, it is crucial to evaluate the biochemical, clinical, and hormonal effects of a combination therapy approach, particularly in patients who have just received a diagnosis. The comparative and synergistic effects of SGLT2 inhibitors in the treatment of early-stage diabetes are gaining attention, even if metformin is still the first-line medication due to its insulin-sensitizing properties. With a focus on biochemical parameters, clinical outcomes, and hormonal changes, the present study aims to evaluate the combined effects of SGLT2 inhibitors and metformin in recently diagnosed patients with type 2 diabetes mellitus.

While the individual metabolic effects of SGLT2 inhibitors and metformin are well established from randomized controlled trials, less is known about their comparative and combination effects specifically in newly diagnosed, treatment-naïve patients in real-world clinical settings where treatment is not randomized. Most existing trials have enrolled patients with established disease or have used strict eligibility criteria that limit generalizability. This study contributes real-world observational data on treatment responses in a clinically important but relatively understudied population—patients at the earliest stage of their disease trajectory. The novelty, therefore, lies not in discovering new drug effects but in characterizing these effects in a previously underrepresented cohort under conditions that reflect actual clinical practice.

## 2. Materials and Methods

### 2.1. Study Design and Setting

This prospective longitudinal study was conducted at the Department of Biochemistry, Baqai Medical University, in collaboration with the Baqai Institute of Diabetology and Endocrinology, after obtaining approval from the Ethics Committee of Baqai Medical University (BMU-EC/01-2022).

### 2.2. Sample Size

Sample size was estimated using standard formulas [11]:

n = Z_1−α/2_^2^ SD^2^/d^2^

where Z_1−α/2_ represents the standard normal variate (1.96 at 5% type I error, *p* < 0.05; 2.58 at 1% type I error, *p* < 0.001), SD is the standard deviation, and d is the absolute precision. The calculated sample size was n = 120. Participants were recruited using a convenience sampling approach; however, efforts were made to minimize selection bias by enrolling patients consecutively during the study period.

### 2.3. Participants and Recruitment Process

Patients were recruited consecutively from the outpatient clinics of the Baqai Institute of Diabetology and Endocrinology. All potentially eligible patients presenting during the specific time frame (March 2022 to March 2023) were screened for inclusion. Inclusion criteria comprised adults (≥18 years) with newly diagnosed type 2 diabetes mellitus (HbA1c > 6.5%) and normal renal function. Exclusion criteria included patients with type 1 diabetes, gestational diabetes, malignancy, bariatric disease, and chronic cardiac or hepatic insufficiency. Written informed consent was obtained from all participants prior to enrollment. A structured questionnaire was used to collect demographic and clinical data.

**Figure 1:**
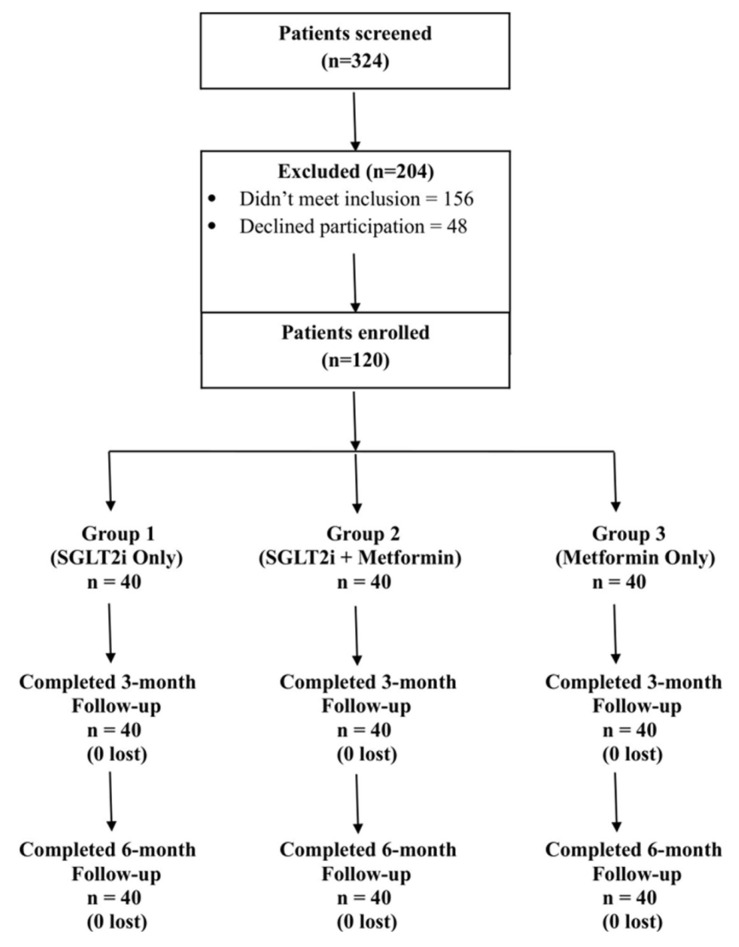
Study flow diagram.

Eligibility was assessed based on predefined inclusion and exclusion criteria by trained clinicians. A total of 324 patients were screened, of whom n = 120 met the eligibility criteria and were included in the final analysis. Patients who did not meet the inclusion criteria or declined participation were excluded. Reasons for exclusion included type 1 and gestational diabetes, any form of malignancy, bariatric disease, and chronic cardiac and hepatic insufficiency. A flow diagram summarizing patient disposition throughout the study is presented in Figure 1.

### 2.4. Group Allocation and Treatment Strategy

Participants were stratified into three groups (n = 40 each): Group 1 (SGLT2 inhibitors only), Group 2 (SGLT2 inhibitors + metformin), and Group 3 (metformin only). Treatment allocation was not randomized; rather, it was based on physician-directed clinical decision-making reflecting real-world practice. The choice of therapy was guided by patient-specific clinical characteristics, glycemic status, comorbidities, and tolerance to medications and these patients were not on any other medication.

*All 120 enrolled participants (100%) completed the full 6-month follow-up period, with no losses to follow-up or missing data at any time point (baseline, 3 months, or 6 months). Consequently, no imputation methods for missing data were required, and all 120 participants were included in the complete-case analysis at each time point.*

This non-randomized allocation introduces the potential for allocation bias and confounding by indication. Therefore, baseline clinical and biochemical characteristics of all groups were recorded and compared to assess group comparability. Patients were followed for six months, with data collection at baseline, 3 months, and 6 months.

Importantly, treatment allocation was not randomized but was based on physician-directed clinical decision-making reflecting real-world practice. This non-randomized allocation introduces the potential for allocation bias and confounding by indication, and the results should therefore be interpreted as hypothesis-generating rather than confirmatory.

### 2.5. Confounding and Baseline Comparability

Given the observational nature of the study and non-randomized treatment allocation, there is a potential risk of confounding by indication. To address this, detailed baseline characteristics, including age, gender, BMI, HbA1c, and relevant comorbidities, were documented for each group.

### 2.6. Clinical and Biochemical Assessment

A Viva Stadiometer from Viva Instruments UK LTD - and Beurer digital scale sourced from Germany were used to record anthropometric and blood pressure data, such as height and weight measured barefoot; BMI was computed as kg/m^2^ (adults) or percentiles (adolescents; WHO standards). In accordance with conventional procedure, waist and hip circumferences were measured, and the waist and hip ratio was computed [12]. According to ACC/AHA 2017 recommendations, blood pressure was taken using a mercury sphygmomanometer, averaging three sitting readings at five-minute intervals [13]. The glucose oxidase-peroxidase technique was used to measure fasting blood glucose levels [14]. Ion-exchange high-performance liquid chromatography (HPLC) was used to test HbA1c [15]. Standard colorimetric techniques were used to evaluate the lipid profile, which included total cholesterol, triglycerides, HDL-C, and LDL-C [16]. Urease and Jaffe’s technique were used to test serum uric acid and creatinine levels, respectively [17]. The CKD-EPI formula was used to determine the estimated glomerular filtration rate (eGFR) [18]. ELISA kits (Waltham, MA, USA) were used to measure serum insulin and glucagon levels in accordance with the manufacturer’s instructions [19]. The Homeostatic Model Assessment for Insulin Resistance (HOMA-IR) was used to calculate insulin resistance [20].

### 2.7. Statistical Analysis

Data were analyzed using IBM SPSS Statistics (version 23.0). Continuous variables were expressed as mean ± standard deviation (SD), and categorical variables were summarized as frequencies and percentages.

Normality of data distribution was assessed using the Shapiro–Wilk test along with visual inspection of histograms and Q–Q plots. All study variables were found to be normally distributed; therefore, parametric tests were applied throughout the analysis, and all continuous variables are presented as mean ± SD. For comparison of baseline characteristics between groups, One-way Analysis of Variance (ANOVA) was used. To evaluate changes over time within and between groups, Repeated Measures ANOVA was employed, with time (baseline, 3 months, and 6 months) as the within-subject factor and treatment group (Group I, II, and III) as the between-subject factor. The time × treatment interaction term was included to assess differences in treatment effects over time. Post hoc analyses were performed using Tukey’s HSD test for between-group comparisons and Bonferroni correction for within-group repeated measures to account for multiple comparisons. Paired comparisons within groups were conducted using the paired *t*-test, where appropriate.

Prior to conducting repeated-measures ANOVA, the underlying assumptions were assessed. Normality of the residuals was verified using the Shapiro–Wilk test at each time point. Sphericity (the assumption that the variances of the differences between all pairs of within-subject time points are equal) was assessed using Mauchly’s test of sphericity. When sphericity was violated (*p* < 0.05 for Mauchly’s test), the Greenhouse–Geisser correction was applied to adjust the degrees of freedom. In this study, sphericity was violated for several outcome variables (including FBS, HbA1c, and HDL cholesterol), and the Greenhouse–Geisser correction was applied accordingly. Homogeneity of between-group variances was assessed using Levene’s test.

No statistical adjustment for confounders was performed; therefore, the potential for residual confounding has been acknowledged as a limitation and considered during the interpretation of the results. A *p*-value < 0.05 was considered statistically significant. Because all participants completed follow-up with no missing data at any time point (see Section 2.4), complete-case analysis was performed without the need for missing data imputation.

## 3. Results

Table 1 shows the comparison of the baseline parameters. It can be observed that no significant difference was found in any parameters across groups at the baseline (*p* > 0.05).

It should be noted that several parameters demonstrated substantial within-group variability, as reflected by relatively large standard deviations. This heterogeneity likely reflects the real-world diversity of newly diagnosed type 2 diabetes patients and may have reduced statistical power to detect modest but clinically meaningful differences between groups.

At the first follow-up, weight, height, and BMI were comparable among the three groups, showing no significant differences (*p* > 0.05). However, waist circumference, hip circumference, and waist-to-hip ratio differed significantly (*p* < 0.01, *p* < 0.01, and *p* = 0.004, respectively), with Group 2 showing lower central adiposity compared to Groups 1 and 3. Glycemic parameters also varied significantly, as fasting blood sugar and HbA1c were higher in Group 1 and lower in Groups 2 and 3 (*p* < 0.01), as shown in Table 2. Among lipid profiles, total cholesterol, triglycerides, and LDL cholesterol were significantly lower in Group 2 (*p* = 0.004, *p* = 0.03, and *p* < 0.01, respectively), while HDL cholesterol remained similar (*p* = 0.718). Renal function markers, including serum creatinine, eGFR and uric acid levels, differed significantly (*p* = 0.001, *p* = 0.008, *p* = <0.01, respectively), and all parameters were lower in Group 2. Group 2 had considerably higher insulin resistance than the other groups, as measured by HOMA-IR (*p* < 0.01). While fasting, insulin levels did not change substantially (*p* = 0.086); glucagon levels were considerably lower in Group 3 compared to Groups 1 and 2 (*p* < 0.01) (Table 2).

Table 3 shows that at the second follow-up, there were no significant differences among the three groups in weight, height, BMI, fasting blood sugar, HbA1c, triglycerides, eGFR, HOMA-IR, or fasting insulin (*p* > 0.05), indicating that overall body size, glycemic control, insulin resistance, and renal filtration were comparable across the treatment regimens. Significant differences were observed in waist circumference, hip circumference, and waist-to-hip ratio (*p* < 0.01, *p* < 0.01, and *p* = 0.001, respectively), with Group 3 showing higher central adiposity compared to Groups 1 and 2. Lipid parameters, including total cholesterol (*p* = 0.002), HDL cholesterol (*p* = 0.011), and LDL cholesterol (*p* < 0.01), also differed significantly, reflecting a more favorable lipid profile in Groups 1 and 2. Renal function markers, serum creatinine (*p* = 0.005), and uric acid (*p* < 0.01) were significantly different, while eGFR remained similar. Glucagon levels were markedly lower in Group 3 compared to Groups 1 and 2 (*p* < 0.01), whereas fasting insulin levels were comparable (*p* = 0.184).

Table 4 shows the statistically significant change in the mean values of all anthropometric, biochemical, and hormonal parameters across the study period from baseline to the second follow-up (*p* < 0.01), except for serum creatinine and estimated glomerular filtration rate (eGFR), which did not show a statistically significant change from baseline to the second follow-up (*p* > 0.05).

Among hormonal parameters, insulin levels showed significant differences across all time points (*p* < 0.05), whereas glucagon levels demonstrated a significant change only between the first follow-up and second follow-up (*p* < 0.01).

## 4. Discussion

The authors acknowledge that the pharmacological effects of SGLT2 inhibitors and metformin individually are well documented. The contribution of this study is not a mechanistic discovery but rather the characterization of treatment responses in a real-world cohort of newly diagnosed, treatment-naïve type 2 diabetes patients—a population that differs substantially from the highly selected participants in randomized trials. Our findings suggest that real-world responses may differ from trial data in certain respects (e.g., the attenuation of differences by six months), highlighting the importance of pragmatic studies.

The present study evaluated the association of metformin, SGLT-2 inhibitors, and their combination with changes in anthropometric, biochemical, and hormonal parameters in newly diagnosed type 2 diabetic patients over a six-month follow-up period. Participants receiving SGLT-2 inhibitor-based therapy, either alone or in combination with metformin, demonstrated greater improvements in anthropometric parameters, including waist circumference, hip circumference, and waist-to-hip ratio, at both follow-up points. These findings are consistent with previous reports suggesting that SGLT-2 inhibitors are associated with reductions in body weight and visceral adiposity, potentially mediated by caloric loss through glycosuria [21]. These findings are supported by previous research, which reported that metabolites of SGLT-2 inhibitors, such as ipragliflozin, were more effective than metformin alone in reducing visceral adiposity [22]. Glycemic parameters, including fasting blood glucose and HbA1c, were reduced at the first follow-up in groups receiving SGLT-2 inhibitors, consistent with clinical trials demonstrating superior glycemic control achieved by SGLT-2 inhibitors, particularly when combined with metformin [23]. By the second follow-up, glycemic control appeared comparable across all groups, indicating that while SGLT-2 inhibitor-based regimens may be associated with earlier improvements, all treatment approaches were linked with improved glycemic outcomes over time.

Lipid profiles, particularly LDL cholesterol and triglycerides, also improved significantly in Group 2 by the first follow-up (*p* < 0.01), consistent with studies showing SGLT2 inhibitors can improve lipid metabolism and reduce cardiovascular risk markers [24]. It was also reported in the previous study that SGLT2 inhibitors reduce “atherogenic index of plasma (AIP)” in patients with type 2 diabetes; thereby, reducing the risk of cardiovascular events in the future [25].

The findings of the study show associations consistent with the protective effect of SGLT2 inhibitors on kidneys, as suggested in the literature, reflected by the significant reduction in uric acid levels by the second follow-up (*p* < 0.01) in Groups 1 and 2. This aligns with the literature suggesting that SGLT2 inhibitors facilitate uricosuria, lowering serum uric acid levels and potentially reducing gout, along with other therapeutic benefits [26]. While serum creatinine levels fluctuated slightly, they remained within the normal range, and no deterioration in eGFR was observed. These findings are directionally consistent with the renal-protective effects of SGLT2 inhibitors reported in large randomized trials such as CREDENCE and DAPA-CKD [27,28]. However, due to the observational nature of our study, we cannot establish causality, and our results should be considered hypothesis-generating rather than confirmatory of renoprotection.

Hormonal analysis revealed a decline in glucagon levels across all groups, with a comparatively greater reduction in the combination therapy group at the second follow-up. Although reductions in insulin levels and HOMA-IR were observed, these changes did not reach statistical significance. These findings may reflect short-term metabolic adaptations, rather than a direct effect of the drug on pancreatic islets [29]. However, further mechanistic studies are required to clarify these observations.

Several limitations must be acknowledged. A key limitation of this study is its non-randomized design, in which treatment allocation was based on physician-directed clinical decision-making rather than random assignment. This may introduce selection bias and confounding by indication, which could influence the observed outcomes. Therefore, the findings should be interpreted as associative rather than causal, and future randomized controlled studies are warranted to validate these results. The sample size was modest, which may have limited the statistical power to detect subtle but clinically meaningful differences across groups. Additionally, dietary intake, physical activity, and other lifestyle factors were not strictly controlled, potentially contributing to variability in biochemical parameters and partially confounding the observed effects. The relatively short duration of follow-up further restricted the ability to assess the long-term sustainability of these improvements, particularly regarding macrovascular and cardiovascular outcomes.

A fundamental limitation of this study is its non-randomized, observational design. Treatment allocation was based on physician-directed clinical decision-making rather than random assignment, which introduces inherent risks of selection bias and confounding by indication. While we statistically compared baseline characteristics across groups and found no significant differences (Table 1, all *p* > 0.05), this does not eliminate the possibility of unmeasured confounding variables that may have influenced both treatment selection and outcomes. Therefore, all observed differences should be interpreted as associations rather than causal effects of the interventions themselves. We explicitly caution readers against inferring associations and potential benefits of any regimen from these data.

The substantial within-group variability observed for several metabolic parameters (evidenced by wide standard deviations) suggests considerable patient heterogeneity that may have obscured true treatment effects or contributed to spurious findings. Future studies with larger sample sizes and more homogeneous patient populations, or alternatively, stratified analyses, based on relevant patient characteristics, would help address this heterogeneity.

Finally, this study does not claim to provide novel mechanistic insights. Readers familiar with the literature will recognize that the observed metabolic effects align with established pharmacology. The value added by this study is the description of these effects in a real-world, non-randomized setting with newly diagnosed patients, which complements the existing evidence base from highly controlled randomized trials. Readers are reminded that the observed reductions in uric acid and stable creatinine levels are associations only; residual confounding (e.g., by dietary changes or unmeasured lifestyle factors) cannot be excluded, and these findings should not be equated with the renoprotective efficacy demonstrated in randomized trials.

## 5. Conclusions

Given the methodological limitations of this observational study (non-randomized design, substantial within-group heterogeneity, and potential confounding by indication), our findings should be considered hypothesis-generating. While we observed associations between certain treatment regimens and specific metabolic parameters, these data do not provide sufficient evidence to conclude the therapeutic superiority of any regimen. The primary value of this study lies in its documentation of real-world treatment responses in newly diagnosed type 2 diabetes patients, which can inform the design of future randomized controlled trials.

The observed associations suggest potential metabolic benefits that warrant further investigation across anthropometric, glycemic, renal, and hormonal parameters. Causal conclusions regarding these potential advantages cannot be drawn from this observational study. Based on the observational design of this study, we cannot conclude causal associations of any treatment regimen. Rather, our findings suggest associations between early combination therapy and certain metabolic improvements, which warrant confirmation through properly powered randomized controlled trials.

## Figures and Tables

**Table 1:** Baseline demographic, anthropometric and clinical parameters.

*Parameters*	*Group*						*p-Value*
	*Group-1*		*Group-2*		*Group-3*		
	*n*	*%*	*n*	*%*	*n*	*%*	
Gender							
Male	21	52.5	22	55.0	25	62.5	0.64
Female	19	47.5	18	45.0	15	37.5	
	***Mean ± SD***		***Mean ± SD***		***Mean ± SD***		***p Value***
Age (years)	52.2 ± 7.2		52.9 ± 8.4		54.4 ± 9.4		0.47
Systolic BP	126.8 ± 14.2		130.2 ± 14.9		126.1 ± 16.3		0.43
Diastolic BP	84.3 ± 6.8		84.5 ± 6.6		83.6 ± 7.5		0.81
Weight (kg)	72.3 ± 13.0		75.8 ± 14.8		76.5 ± 16.6		0.39
Height (cm)	165.8 ± 9.3		161.6 ± 6.6		163.1 ± 10.2		0.10
BMI (kg/m^2^)	26.3 ± 4.1		29.1 ± 5.7		29.1 ± 7.4		0.052
Waist Circumference (cm)	98.5 ± 10.2		98.2 ± 10.5		99.5 ± 9.9		0.91
Hip Circumference (cm)	108.7 ± 9.7		106.7 ± 10.6		107.6 ± 8.0		0.89
WHR	0.9 ± 0.1		0.9 ± 0.2		1.0 ± 0.1		0.067
FBS (mg/dL)	198.4 ± 20.7		200.7 ± 19.8		199.4 ± 18.2		0.94
HbA1c (%)	8.1 ± 0.8		8.6 ± 0.6		8.8 ± 0.6		0.88
Cholesterol (mg/dL)	194.6 ± 41.4		189.6 ± 37.4		197.0 ± 42.6		0.12
Triglyceride (mg/dL)	163.7 ± 66.2		157.7 ± 43.1		180.5 ± 70.3		0.22
HDL (mg/dL)	38.5 ± 4.7		37.1 ± 3.2		37.8 ± 2.8		0.05
LDL (mg/dL)	127.4 ± 28.8		116.3 ± 28.3		121.6 ± 16.5		0.147
Serum Creatinine(mg/dL)	0.9 ± 0.3		0.9 ± 0.2		1.0 ± 0.2		0.118
eGFR	89.9 ± 15.5		92.3 ± 15.1		87.4 ± 16.9		0.395
Uric acid (mg/dL)	7.3 ± 0.7		7.2 ± 0.6		7.2 ± 0.7		0.85
HOMA-IR	21.8 ± 5.3		22.5 ± 5.5		21.2 ± 5.5		0.97
Glucagon (pmol/L)	132.5 ± 17.3		137.7 ± 14.0		137.9 ± 13.7		0.93
Insulin (mU/L)	44.9 ± 9.0		43.4 ± 8.5		43.6 ± 8.9		0.701

*p* < 0.05 was considered statistically significant for Pearson’s and Chi-Square tests and One-Way ANOVA.

**Table 2:** Comparison of anthropometric and clinical parameters at first follow-up.

*Parameters*	*Groups*			*p-Value*
	*Group-1* *Mean* * ± SD*	*Group-2* *Mean* * ± SD*	*Group-3* *Mean* * ± SD*	
Weight (kg)	70.1 ± 12.9	71.7 ± 14.5	74.1 ± 16.5	0.471
Height (cm)	165.8 ± 9.3	161.6 ± 6.6	163.1 ± 10.2	0.103
BMI (kg/m^2^)	25.5 ± 4.0	27.5 ± 5.6	28.2 ± 7.3	0.102
Waist Circumference (cm)	97.7 ± 9.1	86.8 ± 10.7	101.1 ± 10.6	<0.01 *
Hip Circumference (cm)	111.8 ± 9.4	101.5 ± 11.2	104.7 ± 8.1	<0.01 *
WHR	0.9 ± 0.1	0.9 ± 0.2	1.0 ± 0.1	0.004 *
FBS (mg/dL)	196.5 ± 20.8	161.5 ± 16.9	162.4 ± 15.1	<0.01 *
HbA1c (%)	7.6 ± 0.7	7.2 ± 0.1	7.3 ± 0.5	<0.01 *
Cholesterol (mg/dL)	185.9 ± 38.7	156.9 ± 35.2	178.3 ± 43.5	0.004 *
Triglyceride (mg/dL)	151.2 ± 63.3	125.1 ± 38.0	157.4 ± 68.1	0.03 *
HDL (mg/dL)	41.9 ± 4.4	42.5± 3.0	42.4 ± 2.5	0.718
LDL (mg/dL)	120.6 ± 26.7	95.6 ± 26.4	106.9 ± 16.0	<0.01 *
Serum Creatinine (mg/dL)	1.0 ± 0.1	0.9 ± 0.1	1.0 ± 0.1	0.001 *
eGFR	83.7 ± 15.0	82.8 ± 10.5	84.4 ± 13.2	0.008 *
Uric Acid (mg/dL)	6.4 ± 1.7	6.4 ± 0.5	6.9 ± 0.7	<0.01 *
HOMA-IR	16.7 ± 4.5	19.1 ± 4.4	14.5 ± 3.7	<0.01 *
Glucagon (pmol/L)	150.6 ± 14.6	151.0 ± 12.7	103.2 ± 12.5	<0.01 *
Insulin (mU/L)	39.5 ± 7.9	39.3 ± 8.0	36.1 ± 7.2	0.086

* *p* < 0.05 was considered statistically significant for One-Way ANOVA.

**Table 3:** Comparison of anthropometric and clinical parameters at second follow-up.

*Parameters*	*Group*						*p-Value*
	*Group-1*		*Group-2*		*Group-3*		
	*Mean*	*SD*	*Mean*	*SD*	*Mean*	*SD*	
Weight (kg)	68.1	12.8	67.9	14.1	72.9	16.3	0.222
Height (cm)	165.8	9.3	161.6	6.6	163.1	10.2	0.103
BMI (kg/m^2^)	25.0	4.0	26.1	5.4	27.7	7.2	0.111
Waist Circumference (cm)	94.7	8.8	84.2	10.4	99.5	10.4	<0.01 *
Hip Circumference (cm)	110.2	9.3	100.0	11.1	103.6	8.0	<0.01 *
WHR	0.9	0.1	0.9	0.2	1.0	0.1	0.001 *
FBS	151.8	21.7	152.6	13.6	148.1	15.3	0.091
HbA1c	7.1	0.7	7.1	0.5	7.0	0.4	0.490
Cholesterol	179.3	38.3	148.3	35.7	169.5	42.5	0.002 *
Triglyceride	134.2	61.4	116.7	39.0	138.1	57.6	0.168
HDL	44.0	4.4	45.8	2.6	46.2	2.8	0.011 *
LDL	112.2	28.3	87.1	26.5	100.5	16.1	<0.01 *
Serum Creatinine	0.9	0.1	0.9	0.1	1.0	0.1	0.005 *
eGFR	90.8	11.7	90.8	15.4	85.3	12.2	0.100
Uric Acid	5.5	0.7	5.4	0.1	6.5	0.6	<0.01 *
HOMA-IR	12.9	3.8	12.5	3.0	11.6	3.1	0.190
Glucagon	162.3	12.0	148.5	11.9	77.6	9.8	<0.01 *
Insulin	34.1	7.2	32.1	6.6	31.4	6.3	0.184

* *p* < 0.05 was considered statistically significant for One-Way ANOVA.

**Table 4:** Comparison of anthropometric and clinical parameters from baseline to second follow-up.

*Parameters*	*Baseline Mean (SD)*	*F1* *Mean (SD)*	*F2* *Mean (SD)*	*∆* *B vs. F1*	*∆* *B vs.* * F2*	*∆* *F1 vs. F2*
Weight (kg)	74.9 (14.9)	72 (14.7)	69.6 (14.5)	2.87 *	5.22 *	2.349 *
BMI (kg/m^2^)	28.2 (6)	27.1 (5.9)	26.3 (5.8)	1.09 *	1.89 *	0.79 *
WC (cm)	98.2 (11.3)	95.2 (11.8)	92.8 (11.7)	3.05 *	5.40 *	2.35 *
HC (cm)	107.7 (11)	106 (10.5)	104.6 (10.4)	1.64 *	3.06 *	1.42 *
WHR	0.9 (0.1)	0.9 (0.2)	0.9 (0.1)	0.01 *	0.02 *	0.01 *
FBS	198.5 (29)	176.1 (23)	152.1 (17.4)	22.35 *	46.35 *	24.0 *
HbA1c	8.5 (1)	7.8 (0.8)	7.1 (0.5)	0.71 *	1.42 *	0.71 *
Cholesterol	190.4 (41)	173.7 (40.9)	165.7 (40.7)	16.68 *	24.69 *	8.0 *
Triglyceride	167.3 (61.3)	144.5 (59.2)	129.7 (53.9)	22.76 *	37.64 *	14.87 *
HDL	37.5 (3.6)	42.3 (3.4)	45.3 (3.5)	−4.80 *	−7.86 *	−3.06 *
LDL	121.8 (25.4)	107.7 (25.5)	99.9 (26.1)	14.09 *	21.85 *	7.75 *
Serum Creatinine	0.9 (0.2)	0.9 (0.1)	0.9 (0.1)	−0.04 *	−0.01	0.02 *
eGFR	89.9 (15.8)	87 (14.7)	88.9 (13.3)	2.89 *	0.90	−1.98 *
Uric Acid	7.4 (0.9)	6.9 (0.8)	6.2 (0.8)	0.51 *	1.16 *	0.64 *
Glucagon	135.4 (18.4)	134.9 (26.1)	129.5 (38.9)	0.43	5.89	5.45 *
Insulin	44 (8.8)	38.3 (7.8)	32.5 (6.8)	5.70 *	11.46 *	5.75 *
HOMA-IR	21.6 (5.7)	16.8 (4.6)	12.3 (3.3)	4.86 *	9.33 *	4.46 *

B: Baseline, F1: Follow-up-I, F2: Follow-up-II, ∆: Mean Difference. * significant Mean Difference among study participants with *p* < 0.05 using Bonferroni adjustment.

## Data Availability

The original contributions presented in this study are included in the article. Further inquiries can be directed to the corresponding authors.

## Abbreviations

The following abbreviations are used in this manuscript:

CKD	Chronic Kidney Disease
HDL	High-Density Lipoprotein
HOMA-IR	Homeostatic Model Assessment of Insulin Resistance
LDL	Low-Density Lipoprotein
SGLT-2	Sodium-Glucose Co-transporter 2
T2DM	Type 2 Diabetes Mellitus
WHR	Waist-to-Hip Ratio
